# Phloroglucinol-Based Antimicrobial Shape-Memory Photopolymers for Microimprint Lithography

**DOI:** 10.1021/acsomega.4c08277

**Published:** 2024-12-12

**Authors:** Ausrine Pabricaite, Vilte Sereikaite, Aukse Navaruckiene, Vita Raudoniene, Danguole Bridziuviene, Jolita Ostrauskaite

**Affiliations:** † Department of Polymer Chemistry and Technology, Kaunas University of Technology, Radvilenu Rd. 19, 50254 Kaunas, Lithuania; ‡ Biodeterioration Research Laboratory, Nature Research Center, Akademijos Str. 2, 08412 Vilnius, Lithuania

## Abstract

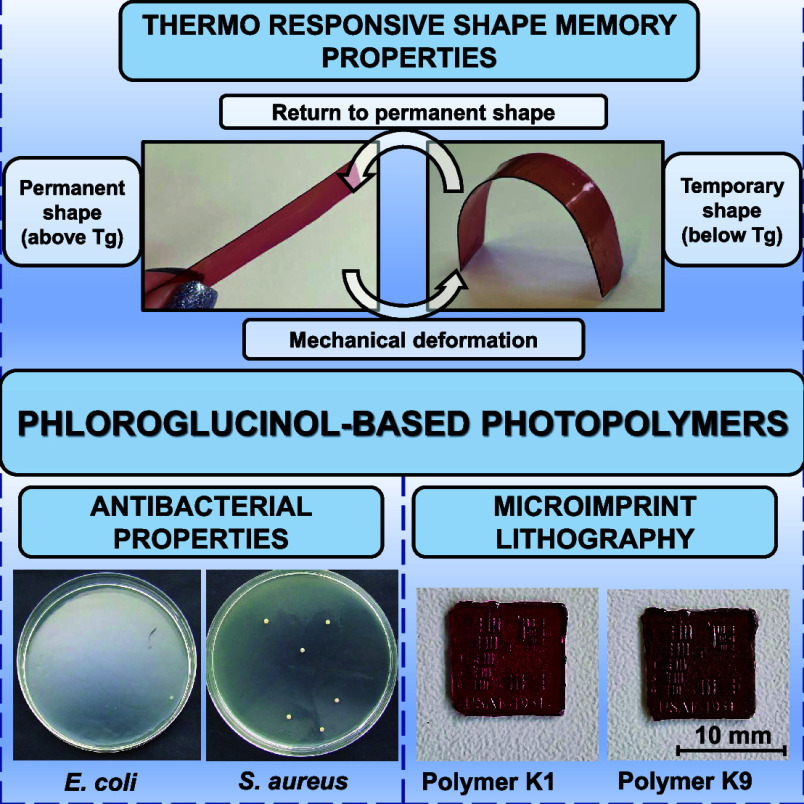

In this study, for the first time, biobased photopolymers were synthesized from phloroglucinol tris epoxy with and without different comonomers, phloroglucinol, 1,4:3,6-dianhydro-D-sorbitol, and 1,4-cyclohexanedimethanol. The rheological, thermal, mechanical, shape-memory, and antimicrobial properties of photopolymers were investigated. The addition of comonomers reduced the photocuring rate (gel time increased from 325 s to 434–861 s) and rigidity (storage modulus decreased from 330.76 to 15.42–85.77 MPa), reduced their brittleness, and increased the flexibility (elongation at break increased from 0.9 to 1.89–4.51%), although the tensile strength of the polymers remained sufficiently high (tensile strength was reduced from 292.00 to 132.62–234.54 MPa). All polymers exhibited a thermoresponsive shape-memory behavior as they could maintain a temporary shape below their glass-transition temperature and return to the permanent shape when the temperature was raised again above the glass-transition temperature. All polymers showed high antibacterial activity against *Staphylococcus aureus* (90.3–96.4%) and **Escherichia coli** (97.8–99.6%) even after 1 h of contact with bacteria. The photoresins were tested in microimprint lithography and confirmed to accurately reproduce the shape features of the 3D printed target. Compositions prepared with 1,4-cyclohexanedimethanol were the most promising due to fast photocuring and the highest flexibility. Synthesized biobased photopolymers have a wide range of properties, making them potential candidates for the production of functional coatings, biomedical devices, or flexible electronics.

## 1 Introduction

Environmental pollution is a growing concern in the world community. One of the major contributors to this concern is the plastic industry, with petroleum-based polymers being a major problem.^1^ Recycling helps reduce environmental pollution; however, it is a temporary solution as materials might lose their unique properties after multiple cycles of recycling (color, transparency, mechanical strength). Furthermore, the resources of fossil fuel are limited and will eventually run out, making biobased polymers a great alternative to fossil-fuel-based polymers.^2^ Even though the interest in replacing synthetic polymers with biobased polymers is growing rapidly, biopolymers accounted for only 1% of the global polymer production in 2023.^3^ Biomass has attracted great attention as a replacement for fossil fuels for the production of biobased materials.^4^ Biobased materials can be obtained in two ways: extracted from natural sources such as living organisms and plants or by chemical modification of renewable raw materials such as vegetable oils, sugars, fats, proteins, and amino acids.^5^

Phloroglucinol (PG) is a phenolic molecule that can be extracted from brown algae, typically found in marine water and rarely in fresh water.^6,7^ Phloroglucinol tris epoxy (PGTE) was successfully tested in thermal polymerization, and the resulting polymers exhibited excellent properties.^8^ PGTE was used with a furan-based curing agent in the synthesis of partially biobased flame-retardant epoxy thermosets.^9^ PGTE was also used in compositions with diglycidyl ether of vanillyl alcohol and disulfide-based diamines for the synthesis of biobased epoxy-amine vitrimers. The obtained thermosets demonstrated properties comparable to those of fossil fuel-based epoxy thermosets.^10^ However, to the best of our knowledge, PGTE has not yet been used in light-induced polymerization.

Photopolymerization is an environmentally friendly and energy-efficient polymerization process.^11^ Its main advantage compared to thermal polymerization is a high curing rate, which allows producing polymers in a few minutes.^12^ Photopolymerization is also suitable for microimprint lithography.^13^ Photocurable resins are molded using transparent molds, usually made of quartz or polydimethylsiloxane, and cured by irradiation through the mold.^14^ The main advantage of microimprint lithography is its fast, easy, and inexpensive but accurate reproduction of various objects from the nanoscale to the macroscale.^15^ Recently, microimprint lithography has attracted great attention due to the increased demand for miniaturized feature sizes as a result of microelectronic development.^16^ The list of materials applicable in microimprint lithography is growing rapidly due to the universality of this method and the need for unique properties of products such as shape-memory behavior,^17^ which allows them to be used in the production of flexible electronic devices.^18^

Shape-memory materials are a part of smart polymeric systems, also known as smart materials.^19^ These materials have the unique ability to change their shape in response to stimuli such as temperature, light, electric or magnetic field, pH, etc.^20,21^ Shape-memory polymers are widely used in the production of sensors, self-assembling structures, electronic devices, and other areas such as biomedicine or aerospace technology.^22^ Recently, various biobased shape-memory polymers were synthesized using thermal polymerization of epoxidized jatropha oil, epoxidized soybean oil with polycaprolactone, epoxidized castor oil with vanillin-furfurylamine, and other materials. The obtained biobased polymers exhibited properties competing with those of synthetic polymers and other unique characteristics such as antimicrobial activity.^23^ Antimicrobial materials are important as they can prevent the spread of diseases as well as prevent food from spoiling for a longer period.^24^ Most hospital-acquired infections occur due to microbial growth on medical devices and can be easily prevented by using antimicrobial materials.^25^ Antimicrobial polymers can be used in the production of functional antimicrobial coatings. The most advantageous approach to the production of antimicrobial polymers is the chemical modification of polymers by incorporation of antimicrobial moieties.^26^ However, only a few biobased antimicrobial shape-memory polymers were obtained by photopolymerization.^27,28^

In this work, for the first time, the biobased compound PGTE was chosen as the main monomer for the synthesis of photopolymers as it has already shown promising results in thermal polymerization, with polymer properties similar to those of fossil fuel-based polymers.^10^ Three different comonomers, PG that can be produced from citrus peel,^29^ 1,4:3,6-dianhydro-D-sorbitol (ISO) produced from wheat,^30^ and 1,4-cyclohexanedimethanol (CHDM) produced from biomass^31^ in different ratios (10, 20, and 30%), were used in this study in order to increase the flexibility and decrease the brittleness of the resulting polymers. [4-(1-Methylethyl)phenyl](4-methylphenyl)iodonium trifluorotris(1,1,2,2,2-pentafluoroethyl)phosphate (IK-1) was used as a photoinitiator as it exhibits high reactivity and excellent solubility in various solvents and monomers. The obtained polymers have a wide range of thermal, mechanical, and antimicrobial properties. Flexibility and nonbrittleness are crucial for the production of flexible objects or devices;^18^ an increase in the amount of comonomers resulted in increased flexibility and lowered the rigidity of polymers, suggesting their suitability for this application. Microimprint lithography is a fast and accurate method for the production of microelectronics; all polymers showed promising results in this application.

## 2 Materials and Methods

### 2.1 Materials

**Figure 1:**
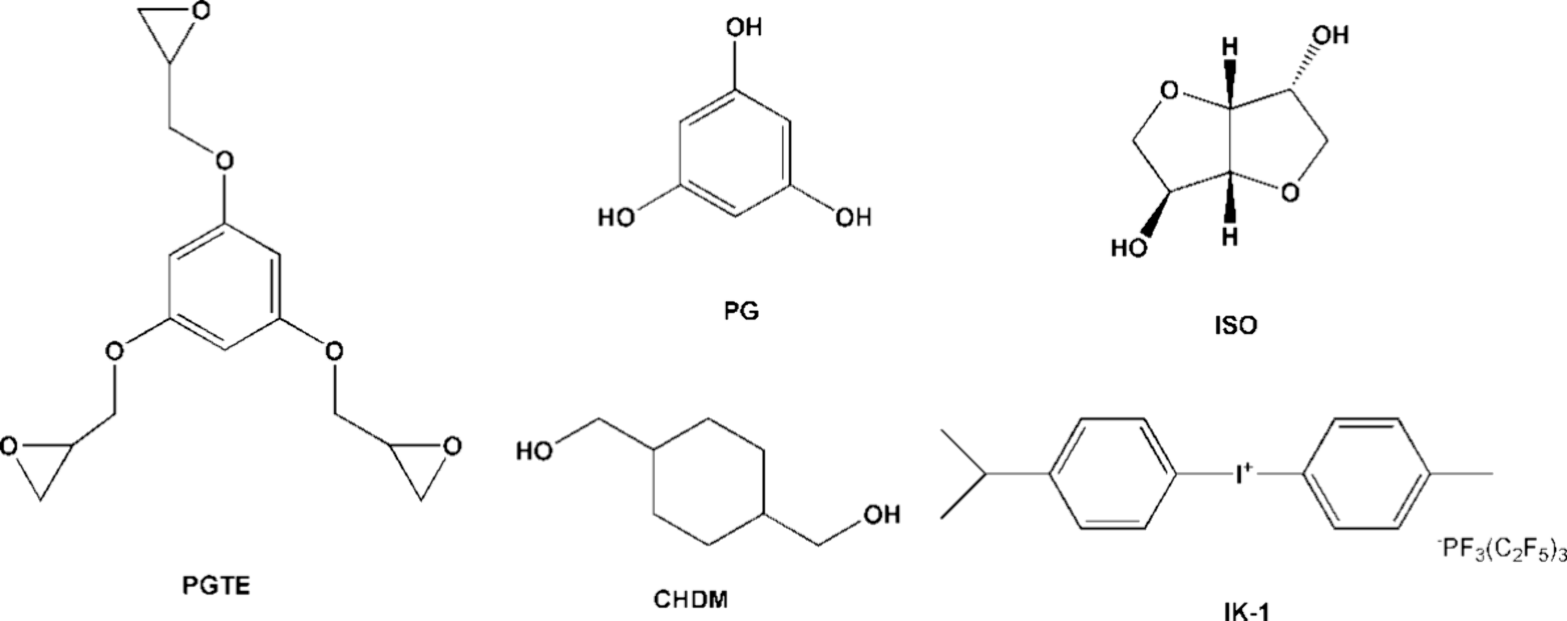
Chemical structures of PGTE, PG, ISO, CHDM, and IK-1.

PGTE (99%, Specific Polymers), PG (99%, Merck), ISO (98%, Merck), CHDM (99%, Merck), and IK-1 (99%, San-Apro Ltd.) (Figure 1) were used as received.

### 2.2 Preparation of Photopolymer Specimens

The initial mixtures containing 70–100 mol % PGTE, 0–30 mol % comonomer (FG, ISO, or CHDM), 3 mol % of the photoinitiator IK-1, and 0.1 g of THF were stirred with a magnetic stirrer at 45 °C for 5 min in a 20 mL glass container until the foggy liquid with visible crystals of the photoinitiator became transparent with no visible crystals or other solid particles. A homogeneous resin was poured into a Teflon mold and cured for 35–40 min in a UV/vis irradiation chamber, whose characteristics have been described in a previous publication.^32^ The compositions of the resins are listed in Table 1.

**Table 1:** Compositions of Resins **K0–K9**

resin	amount of PGTE, mol %	comonomer	amount of comonomer, mol %	amount of photoinitiator IK-1, mol %
**K0**	100			3
**K1**	90	PG	10	3
**K2**	80	PG	20	3
**K3**	70	PG	30	3
**K4**	90	ISO	10	3
**K5**	80	ISO	20	3
**K6**	70	ISO	30	3
**K7**	90	CHDM	10	3
**K8**	80	CHDM	20	3
**K9**	70	CHDM	30	3

**Figure 2:**
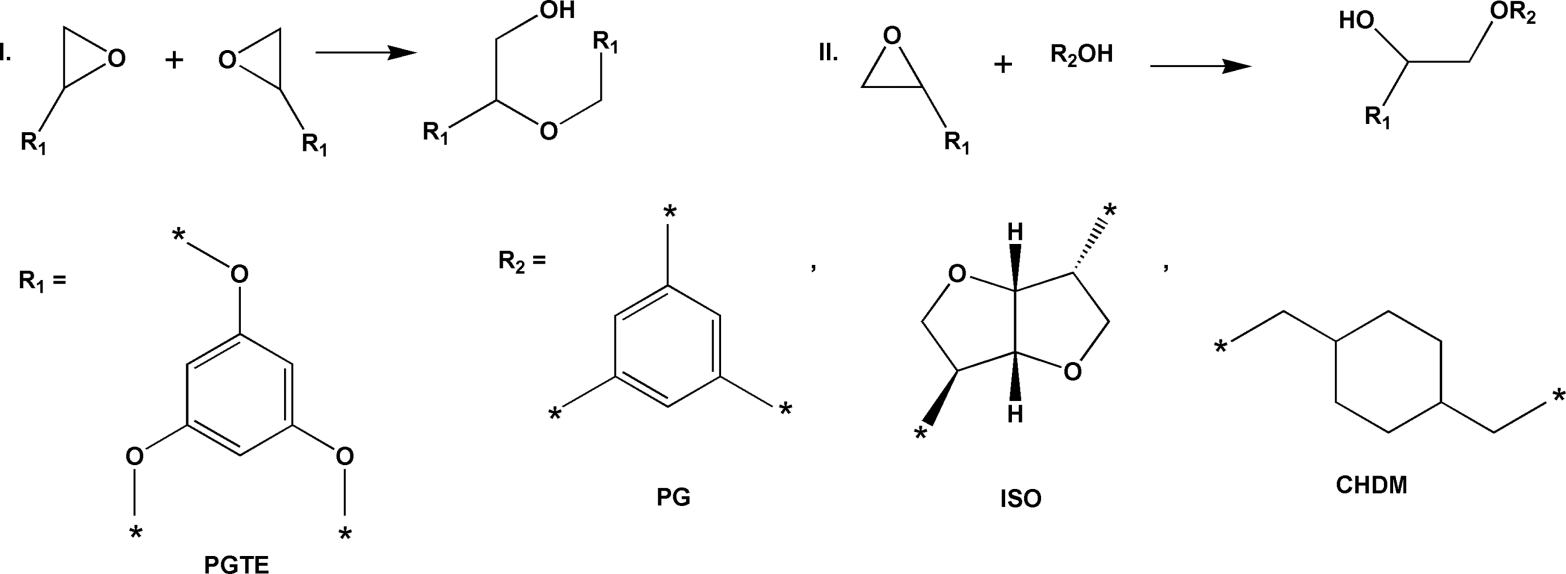
Scheme of homopolymerization of the monomer containing epoxy groups (I) and scheme of copolymerization of monomers containing epoxy and hydroxy groups (II).

During the photocuring of PGTE-based resins, homopolymerization of the monomer containing epoxy groups takes place (Figure 2I), as well as copolymerization of monomers containing epoxy and hydroxy groups (Figure 2II).^32^ The reaction mechanism is cationic.

### 2.3 Characterization Techniques

Fourier transformation infrared spectroscopy (FT-IR) spectra were recorded, and a Soxhlet extraction and swelling test were performed for the characterization of the polymer structure. Dynamic mechanical thermal analysis (DMTA), dynamic mechanical analysis (DMA), and thermogravimetric analysis (TGA) were used for the investigation of the thermal properties of polymers. The shear mode with a shear strain of 0.1%, a frequency of 1 Hz, and a normal force of 5 N was used during DMA and DMTA. The tensile test was used for the investigation of the mechanical properties. Tensile strength, elongation at break, and Young’s modulus were determined. The methodology of these experiments was described in a previous publication.^33^

The UV/vis cure tests of the resins were performed on an MCR302 rheometer (Anton Paar) using the methodology described in a previous publication.^26^

Antimicrobial tests of polymer films were performed using the methodology described in a previous publication.^26^ The final inoculum concentrations were 5.0 × 10^6^ colony-forming units/mL (CFU/mL) for *Staphylococcus aureus* (*S. aureus*), 2.0 × 10^6^ for **Escherichia coli** (*E. coli*), 1.0 × 10^5^ for *Aspergillus flavus* (*A. flavus*), and 1.5 × 10^5^ for *Aspergillus niger* (*A. niger*).

The data collected during the tests listed above were statistically analyzed using the Microsoft Excel program ANOVA.

The suitability of the resins for replica production was tested by microimprint lithography^34^ using a methodology described in a previous publication.^32^ The UV irradiation chamber was used to cure the resins through the soft mold and obtain photocured replicas. The curing time was set to 35 min.

## 3 Results and Discussion

### 3.1 Photocuring Kinetics

**Figure 3:**
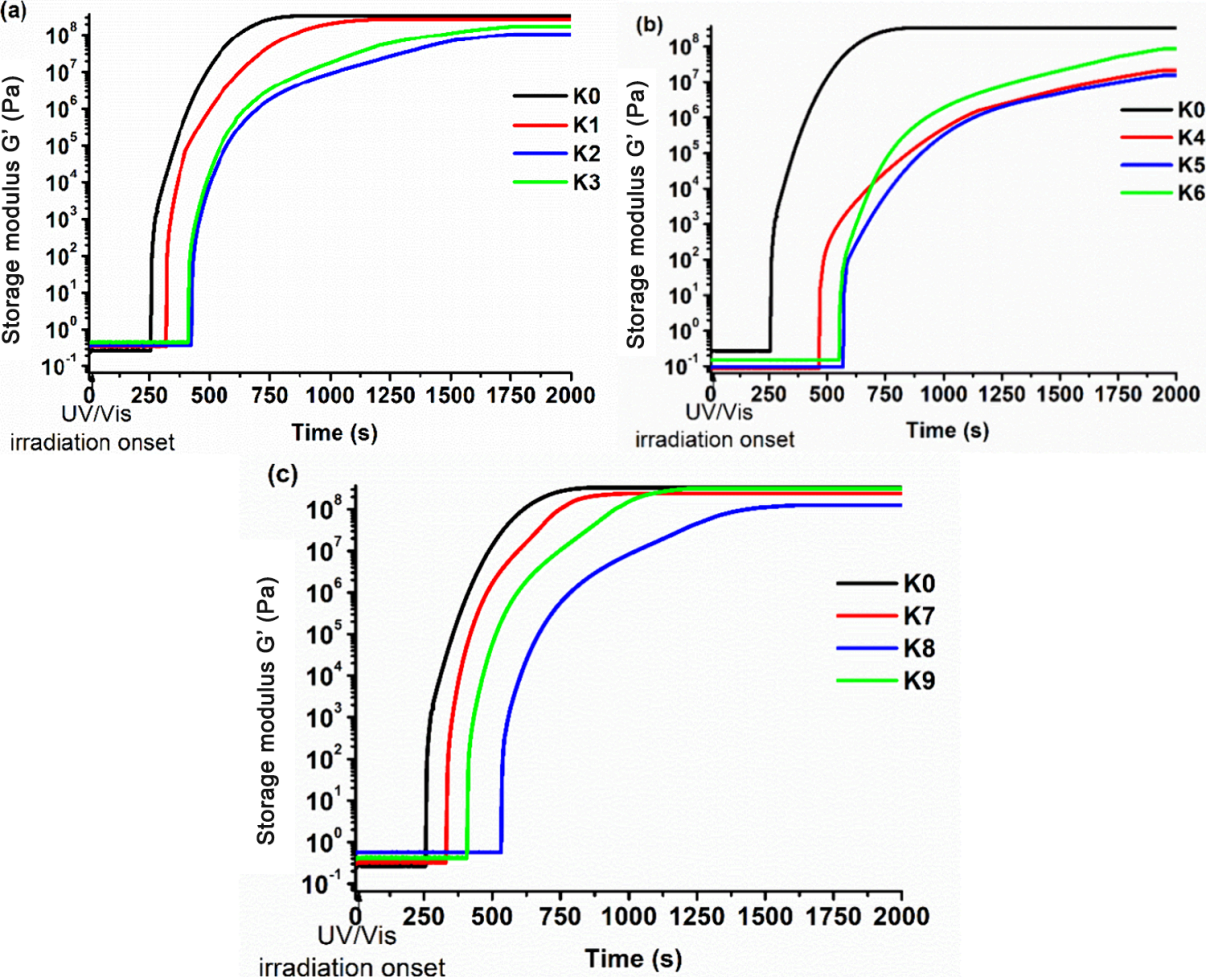
Dependence of the storage modulus of the resin **K0**, PG-containing resins **K1–K3** (a), **K0** and ISO-containing resins **K4–K6** (b), and **K0** and CHDM-containing resins **K7–K9** (c) on irradiation time.

The photocuring kinetics of PGTE-based resins was investigated by real-time photorheometry. The results are summarized in Table 2. The dependence of the storage modulus of the resins on the irradiation time is shown in Figure 3.

**Table 2:** Rheological Characteristics of Resins **K0–K9**

resin	storage modulus *G*‘, MPa	loss modulus *G*′′, MPa	complex viscosity η, MPa s	induction period, s	gel point, *t*_gel_, s
**K0**	330.76 ± 12.03	170.55 ± 7.52	3.45 ± 0.10	250 ± 5	325 ± 5
**K1**	270.31 ± 7.24	123.29 ± 5.17	3.54 ± 0.09	346 ± 6	435 ± 6
**K2**	106.91 ± 4.97	65.08 ± 2.43	1.99 ± 0.03	424 ± 4	585 ± 4
**K3**	174.47 ± 5.68	85.60 ± 3.25	3.07 ± 0.05	420 ± 7	560 ± 7
**K4**	21.42 ± 0.98	7.04 ± 0.25	0.36 ± 0.00	480 ± 8	847 ± 8
**K5**	15.42 ± 0.61	6.33 ± 0.13	0.34 ± 0.00	570 ± 9	861 ± 9
**K6**	85.77 ± 1.43	54.82 ± 1.74	1.66 ± 0.03	560 ± 9	852 ± 9
**K7**	239.97 ± 7.04	44.77 ± 2.13	2.06 ± 0.08	315 ± 6	434 ± 6
**K8**	124.43 ± 6.02	36.28 ± 1.14	5.18 ± 0.18	520 ± 8	631 ± 8
**K9**	314.01 ± 10.32	86.35 ± 3.17	3.89 ± 0.09	402 ± 5	525 ± 5

The most rigid polymer was the neat PGTE polymer **K0,** which showed the highest value of the storage modulus (330.76 MPa) and the fastest photocuring (*t*_gel_ = 325 s). The lowest rigidity and photocuring rate were observed when the comonomer ISO was used in the composition. In these cases, the storage modulus was in the range of 15.42–85.77 MPa and *t*_gel_ was in the range of 847–861 s. Despite the high molecular stiffness of ISO provided by the ring structure,^35^ the resulting PGTE-based copolymers do not exhibit high stiffness as only one hydroxyl group of ISO can participate in the reactions due to its spatial position,^36^ and ISO becomes a side moiety of the polymer chain. When the amount of comonomer ISO was increased from 0% (**K0**) to 10% (**K4**), the values of *G*′ reduced from 330.76 to 21.42 MPa and the *t*_gel_ increased from 325 to 847 s because its fragments prevented the polymer chains from packing more tightly. However, when the amount of ISO was increased to 30% (**K6**), the value of *G*′ increased up to 85.77 MPa and the value of *t*_gel_ decreased slightly (852 s) compared to the composition with 20% ISO (**K5**). The reason for that might be spatial hindrances, which are the result of the denser cross-linked structure formed due to PGTE homopolymerization^37^ and the total amount of hydroxyl groups spatially available for polymerization in the system. When the amount of ISO was increased from 0 to 20%, the amount of functional hydroxyl groups also increased, leading to PGTE and ISO copolymerization. However, the amount of hydroxyl groups was too low and not all of them were able to react with the epoxy groups of PGTE because of the ISO structure.^36^ When the concentration of ISO was increased to 30%, the concentration of hydroxyl groups in the resin also increased and made it easier for hydroxyl groups of ISO to find and react with the epoxy groups of PGTE in the resin, resulting in a more rigid polymer and faster photocuring. The same effect was observed in the values of the induction period as its value increased with the increase of ISO amount from 0 to 20% and then slightly decreased when the ISO amount was 30%.

The resins with PG showed the same tendency of rigidity and photocuring rate change with the change of PG concentration as ISO. The addition of PG slowed down photopolymerization probably due to the lower flexibility of the aromatic fragments. However, the rigidity did not increase with the addition of PG. The reason for that might be the higher amount of hydroxyl groups in the structure of PG. During polymerization, they can participate in the formation of strong hydrogen bonds with the released protons, leading to a decrease in the effective proton concentration and inhibition of polymerization.^38^

The highest values of the storage modulus were obtained when CHDM was used in the composition compared to those with PG and ISO. However, there was the same tendency of rigidity and photocuring rate as those with the samples containing ISO and PG. CHDM has a cyclic aliphatic structure and can be used as a plasticizer.^39^ A lower amount of hydroxyl groups (compared to PG) limits the formation of hydrogen bonds and inhibition of polymerization, which results in lower values of *t*_gel_ and higher values of the storage modulus. For example, the PG-containing resin **K3** reached the rigidity of 174.47 MPa and the value of *t*_gel_ was 580 s, while the CHDM-containing resin **K9** reached the rigidity of 314.01 MPa and the value of *t*_gel_ was 525 s.

### 3.2 Characterization of Polymer Structure

**Figure 4:**
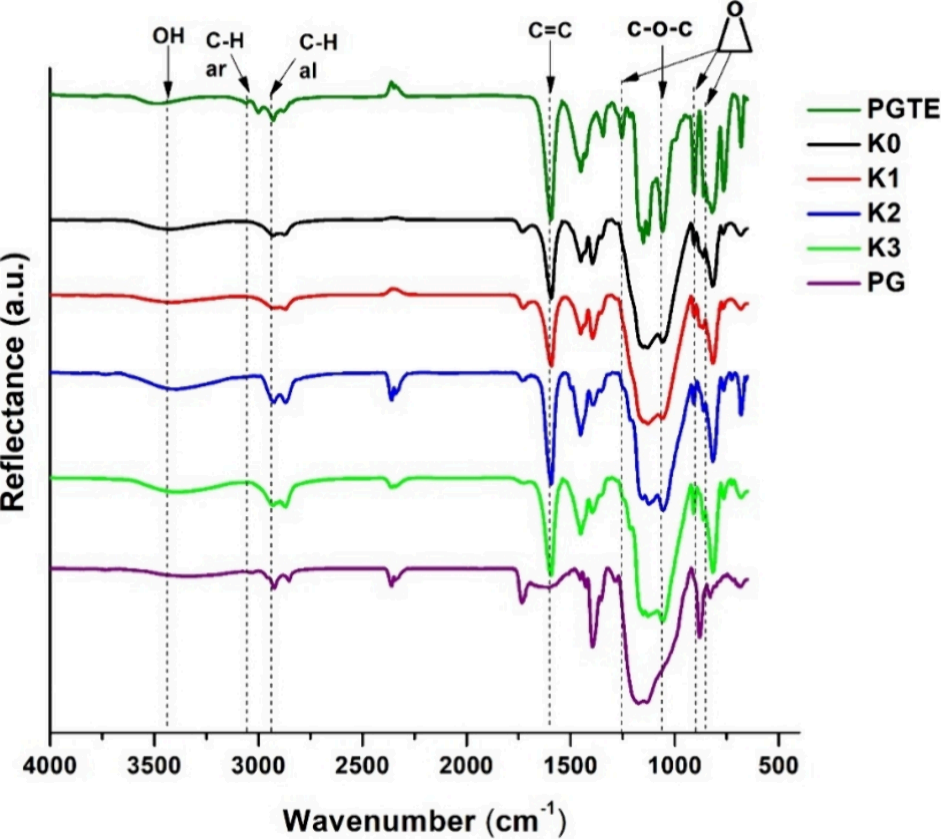
FT-IR spectra of PGTE, PG, and the polymers **K0–K3**.

The chemical structure of the polymers was confirmed by FT-IR spectroscopy. The signals of the epoxy group that were present at 861, 907, and 1253 cm^–1^ in the spectra of PGTE were reduced in the polymer spectra, indicating that the polymer was formed during photocuring; however, not all functional groups reacted due to the spatial hindrances. The other characteristic group signals were also visible in the spectra: OH at 3392–3486 cm^–1^, C–O–C at 1059–1125 cm^–1^, aromatic C–H at 3042–3096 cm^–1^, and aliphatic C–H at 2927–2946 cm^–1^. As an example, the FT-IR spectra of PGTE, PG, and the photopolymers **K0**–**K3** are presented in Figure 4.

The Soxhlet extraction and swelling tests were performed to confirm the cross-linked structure of polymers **K0–K9**. The yield of the insoluble fraction was in the range of 77–95% (Table 3). The neat PGTE polymer **K0** has one of the highest values of the yield of insoluble fraction (93%) and the lowest swelling values in acetone (2.4%), indicating that a dense polymer structure with short chains between cross-linking points was formed and a low amount of the linear or branched polymer fragments remained after photocuring. Polymers **K3**, **K6,** and **K9**, prepared with 30% comonomers, have the highest values of the yield of insoluble fraction and the lowest swelling values, while polymers **K2**, **K5,** and **K8**, prepared with 20% comonomers, have the lowest values of insoluble fraction and the highest swelling values.

**Table 3:** Characteristics of Polymers

polymer	yield of insoluble fraction, %	swelling value in acetone, %	*T*_dec-10%_, °C	*T*_g_, °C
**K0**	93	2.4	334	62
**K1**	88	4.1	287	47
**K2**	85	9.3	276	43
**K3**	91	3.4	309	44
**K4**	78	7.7	280	33
**K5**	77	10.0	286	32
**K6**	84	4.5	287	38
**K7**	89	4.5	327	49
**K8**	80	9.0	305	47
**K9**	95	3.7	331	52

Polymers prepared with ISO had the lowest values of the yield of insoluble fraction and the highest swelling in acetone values. The reason is that ISO leads to the formation of branched polymer fragments and longer chains between the cross-linking points in the polymer structure.^36^ Polymers prepared with CHDM and PG had similar values of the yield of insoluble fraction and swelling in acetone. For example, the swelling value of the PG-containing polymer **K1** was 4.1%, while the swelling value of the CHDM-containing polymer **K7** was 4.5%. The reason for this is the shorter chains formed between the cross-linking points in the polymer structure.

### 3.3 Thermal Properties of Polymers

**Figure 5:**
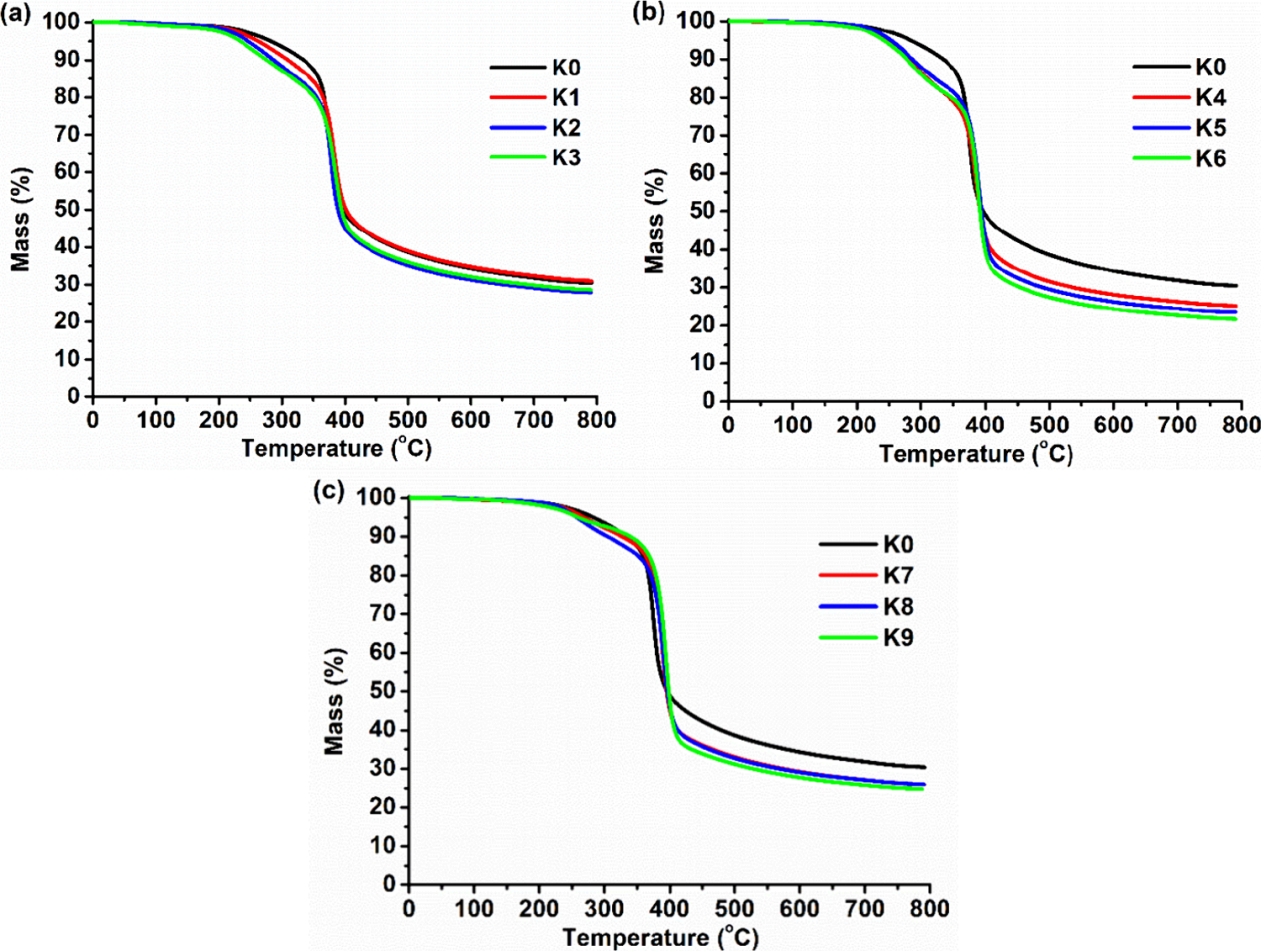
Thermogravimetric curves of the polymer **K0** and PG-containing polymers **K1–K3** (a), **K0** and ISO-containing polymers **K4–K6** (b), and **K0** and CHDM-containing polymers **K7–K9** (c).

TGA and DMTA were selected to determine the thermal characteristics of polymers **K0–K9**. The results are summarized in Table 3. The thermal decomposition of the polymers occurred in one or two steps (Figure 5). The thermal decomposition of the neat PGTE polymer **K0** occurred in one step, while the thermal decomposition of polymers containing PG, ISO, and CHDM (**K1–K9**) fragments occurred in two steps. The reason for this might be the presence of a certain amount of low-molecular-weight compounds and/or oligomers that were not incorporated into the cross-linked structure of polymers **K1–K9**, which thermally decompose easier than the cross-linked parts of these polymers. The neat PGTE polymer **K0** had the highest thermal stability (*T*_dec-10%_ = 334 °C) due to its rigid structure and short chains between cross-linking points. The lowest thermal stability was shown by polymers prepared with ISO (*T*_dec-10%_ = 280–287 °C). Polymers prepared with CHDM showed high thermal stability (*T*_dec-10%_ = 305–331 °C). These results correlate with the values of the yield of the insoluble fraction. Overall, polymers **K0–K9** showed high thermal stability (*T*_dec-10%_ = 276–334 °C) that was similar to glycerol diglycidyl ether-based photopolymers (*T*_dec-10%_ = 270–343 °C)^40^ and higher than that of vanillin alcohol diglycidyl ether-based photopolymers (*T*_dec-10%_ = 227–274 °C), which were considered as alternatives to petroleum-derived films, coatings, or 3D printed objects.^41^

**Figure 6:**
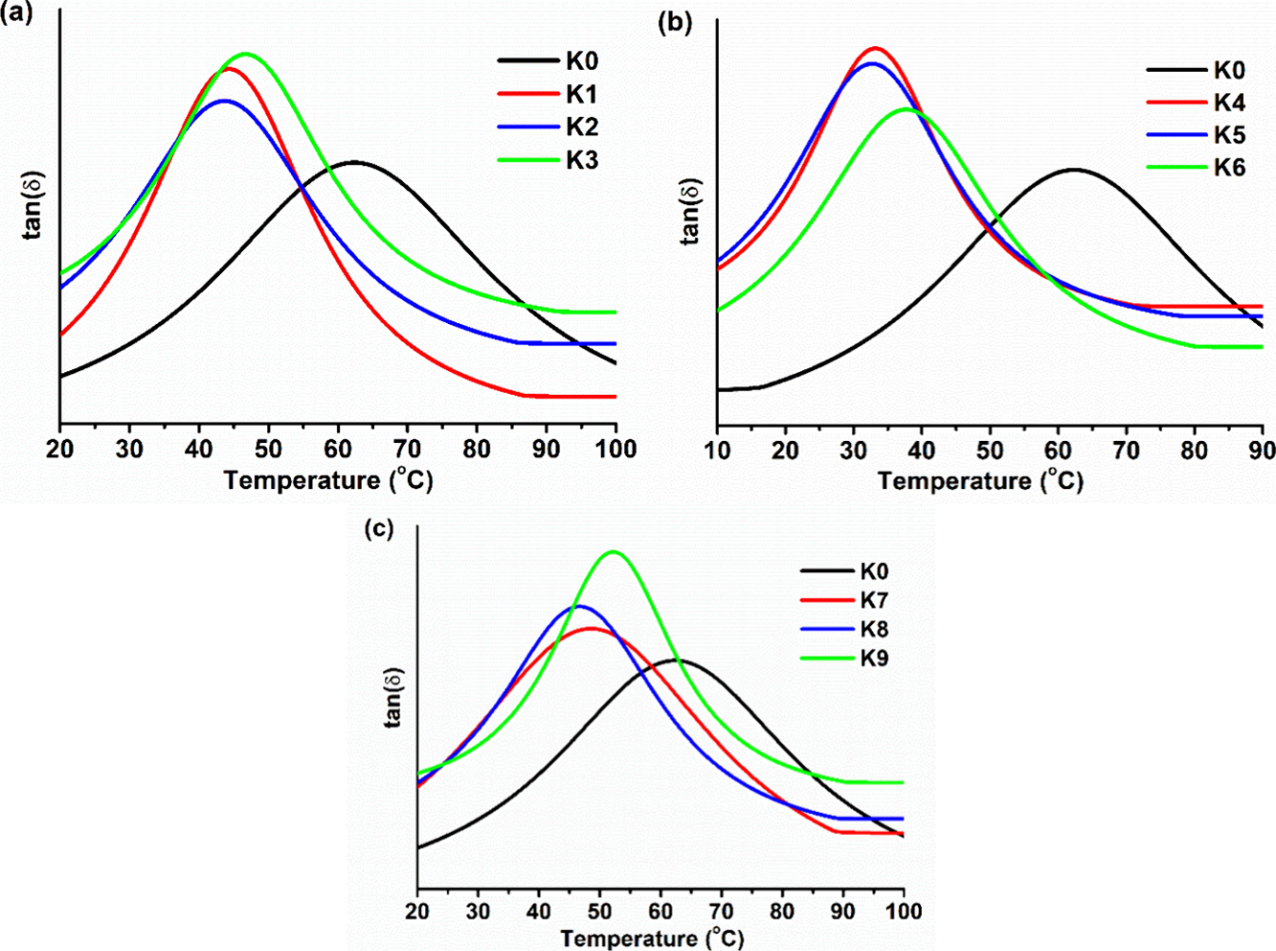
DMTA curves of polymer **K0** and PG-containing polymers **K1–K3** (a), **K0** and ISO-containing polymers **K4–K6** (b), and **K0** and CHDM-containing polymers **K7–K9** (c).

The glass-transition temperature (*T*_g_) of the polymers was in the range of 33–62 °C. The wide *T*_g_ range expands the application areas of polymers as it is an important characteristic for selecting the most suitable polymer for the desired application. The DMTA curves are presented in Figure 6. The highest *T*_g_ was shown by the polymer **K0** (*T*_g_ = 62 °C), which was prepared without comonomers. In all cases, polymers prepared with 20% comonomers (**K2**, **K5**, and **K8**) had the lowest values of *T*_g_ within their groups, while polymers prepared with 30% comonomers (**K3**, **K6**, and **K9**) had the highest values within their groups. The reason for that might be the shorter chains formed between cross-linking points in the structure of polymers **K3**, **K6**, and **K9**, which is confirmed by their low values of swelling in acetone. *T*_g_ values of all polymers were higher than or similar to those of oleic acid-based photopolymers (*T*_g_ −48–33 °C) that were considered suitable materials for coatings or adhesives.^42^

### 3.4 Mechanical Characteristics of Polymers

**Figure 7:**
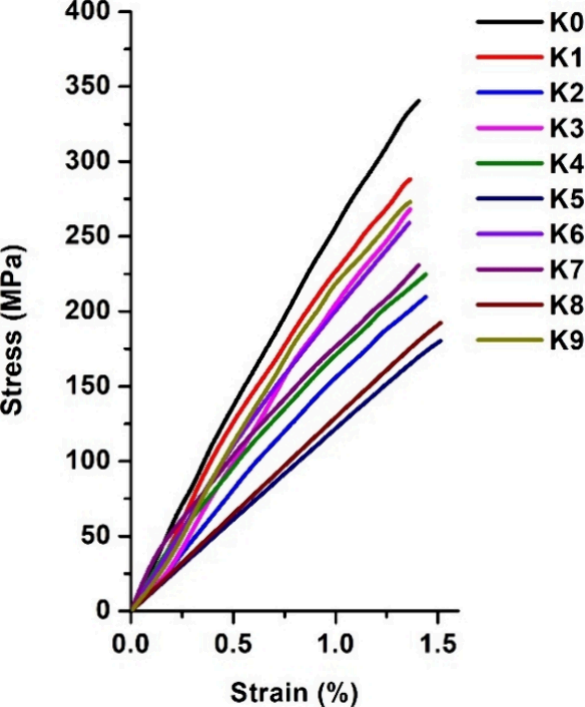
Tensile stress–strain curves of polymers **K0–K9**.

A tensile test was performed to determine the mechanical characteristics of the polymers. The results are presented in Table 4. The stress–strain curves are presented in Figure 7. All polymers showed high values of Young’s modulus (20.05–14.21 GPa) and tensile strength (132.62–292.00 MPa) and low values of elongation at break (0.90–4.51%). The polymer **K0** has an extremely high tensile strength, which is caused by its high cross-linking density, the presence of aromatic structural fragments, and short aliphatic chains. Polymers prepared with 10 and 20% comonomers showed the lowest values of Young’s modulus and tensile strength and the highest values of elongation at break. The reason for that was the longer chains formed between cross-linking points in polymers, confirmed by the higher swelling values. Correlating to the results of the yield of insoluble fraction, the highest values of Young’s modulus and tensile strength were shown by the neat PGTE polymer **K0** (20.05 GPa and 292.00 MPa) and the polymers prepared with 30% comonomers: PG-containing polymer **K3** (17.96 GPa and 194.05 MPa), ISO-containing polymer **K6** (16.92 GPa and 168.11 MPa), and CHDM-containing polymer **K9** (18.37 GPa and 234.54 MPa). Polymers prepared with PG showed the highest values of Young’s modulus and tensile strength and the lowest elongation at break values due to the aromatic structure of PG and shorter chains between the cross-linking points. Slightly lower or similar Young’s modulus and tensile strength values compared to polymers prepared with PG were shown by polymers containing CHDM fragments. The reason for this was the short chains formed between the cross-linking points in the polymer structures, as was confirmed by the swelling test. However, the elongation-at-break values of polymers prepared with CHDM were higher due to the flexible aliphatic chains in the CHDM structure. Polymers prepared with ISO possessed the lowest values of Young’s modulus and tensile strength among the groups due to the spatial position of the hydroxyl group of ISO, which cannot participate in photopolymerization, and ISO becomes a side moiety of the polymer chain. All polymers showed a higher tensile strength and slightly lower elongation-at-break values compared to those of the bisphenol A-based polymer (67.6 MPa and 5.4%), whose mechanical characteristics were suitable for the production of electronic devices.^43^ PGTE-based polymers also showed higher tensile strength than epoxidized soybean oil-based shape-memory polymers (4.5–17.5 MPa), which were successfully used as orthopedic plasters.^44^ However, due to the lower *T*_g_ polymers, K4–K6 are not suitable for production of plasters, while other PGTE-based polymers are suitable candidates for such applications. Furthermore, Young’s modulus values of all PGTE-based polymers were higher than the LY-556 epoxy composites (1.5–3.6 GPa), which were used for biomedical applications.^45^

**Table 4:** Mechanical Characteristics of Polymers

polymer	Young’s modulus, GPa	tensile strength, MPa	elongation at break, %
**K0**	20.05 ± 0.09	292.00 ± 9.23	0.90 ± 0.03
**K1**	18.23 ± 0.08	197.23 ± 7.54	1.89 ± 0.04
**K2**	16.06 ± 0.01	153.86 ± 2.23	2.19 ± 0.04
**K3**	17.96 ± 0.07	194.05 ± 7.90	2.09 ± 0.02
**K4**	16.24 ± 0.07	159.39 ± 1.45	2.15 ± 0.03
**K5**	14.21 ± 0.01	145.20 ± 2.40	3.42 ± 0.04
**K6**	16.92 ± 0.03	168.11 ± 6.93	3.09 ± 0.06
**K7**	16.26 ± 0.05	173.23 ± 8.01	3.84 ± 0.04
**K8**	14.84 ± 0.04	132.62 ± 1.03	4.51 ± 0.05
**K9**	18.17 ± 0.09	234.54 ± 8.64	4.13 ± 0.02

### 3.5 Shape-Memory Properties of Polymers

**Figure 8:**
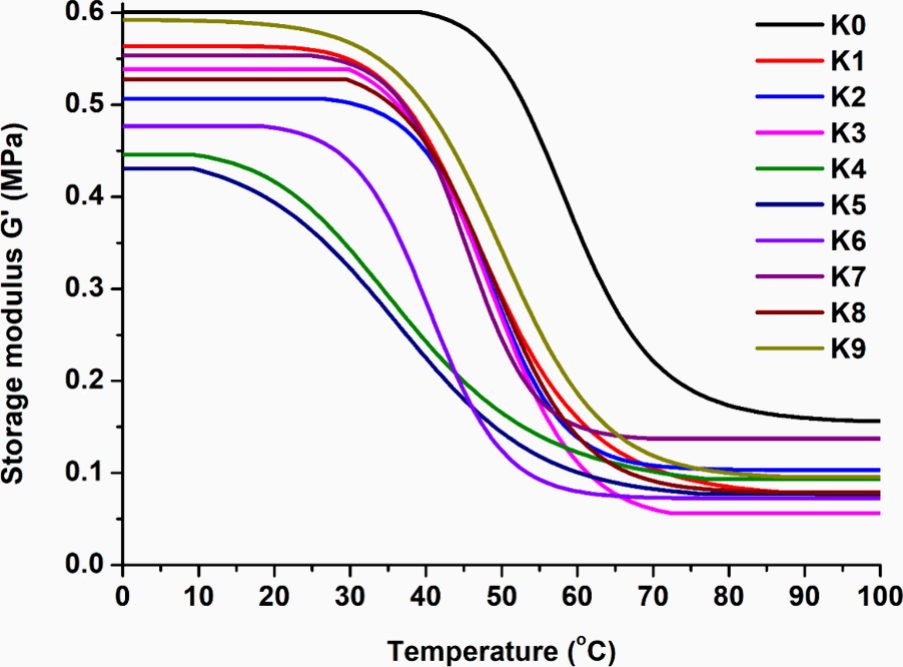
Dependency of storage modulus *G*′ of polymers **K0–K9** on temperature.

Thermoresponsive shape-memory properties of polymers **K0–K9** were determined by *T*_g_.^46^ Dynamical mechanical analysis was performed to characterize shape-memory/mechanical properties of the developed polymers. The dependence of storage modulus *G*′ on temperature is presented in Figure 8. The measurement was carried out until a plateau in the curve was reached, which indicated that no changes were occurring in the polymer structure. The chosen temperatures were 0 °C (before *T*_g_) and 100 °C (after *T*_g_). The storage modulus of polymers decreased as the temperature increased to values higher than those of their *T*_g_. As a result, the PGTE-based polymers were flexible and soft at temperatures above their *T*_g_ and became rigid when the temperature decreased below their *T*_g_.

**Figure 9:**
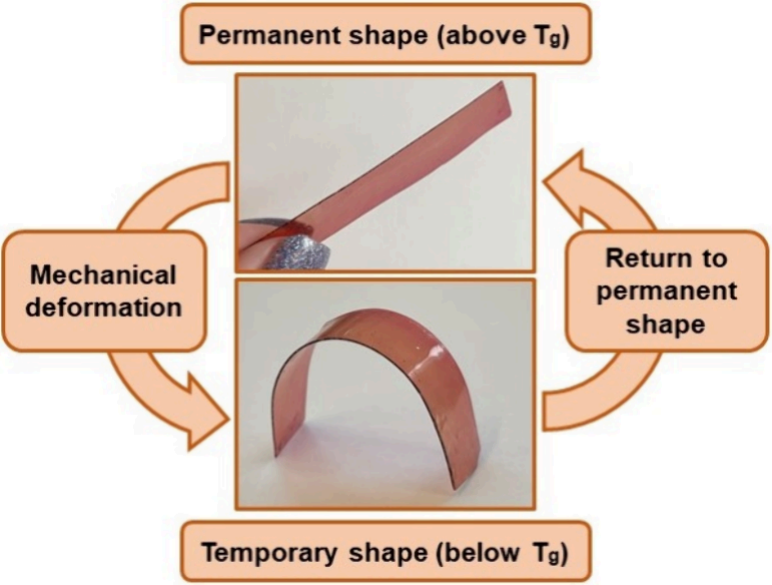
Scheme of studies of shape-memory properties of the polymer **K1**.

All polymers exhibited thermally responsive shape-memory properties, described in more detail in our previous publication.^26^ As an example, the scheme of the heating–cooling–heating cycles of polymer **K1** is presented in Figure 9. PGTE-based polymers have flexible aliphatic fragments, which allow them to change their permanent shape when heated above the *T*_g_, and stiff aromatic fragments, which allow them to maintain their shape. These fragments are responsible for the shape-memory properties of polymers. These results show that the obtained polymers are suitable for application as smart materials due to their ability to change shape by a change in temperature. Also, the wide range of *T*_g_ expands the application areas of polymers as it is a very important characteristic for most applications.

### 3.6 Antimicrobial Properties

The antifungal and antibacterial activity of the polymer films was investigated after 1 h of contact of bacteria or fungi with the polymer specimens. Such a short contact time compared to the standard one, which is 24 h, and 10 times higher than the standard concentration of colony-forming units/mL of fungi and bacteria (ISO 22196:2011^47^ and ISO 16869:2008^48^) were selected to prove that PGTE-based polymers are highly antimicrobial and can reduce microbial spore viability in a short period of time. The obtained results are presented in Table 5.

**Table 5:** Antimicrobial Characteristics of Polymers

polymer	reduction in microbial spores (CFU/mL) after 1 h of contact, %			
	*Escherichia coli*	*Staphylococcus aureus*	*Aspergillus flavus*	*Aspergillus niger*
**K0**	99.2 ± 0.1	96.4 ± 0.3	11.0 ± 0.0	8.7 ± 0.1
**K1**	98.1 ± 0.2	90.3 ± 0.0	11.3 ± 0.1	11.6 ± 0.2
**K2**	97.8 ± 0.3	90.4 ± 0.0	13.2 ± 0.2	13.3 ± 0.1
**K3**	96.2 ± 0.2	90.3 ± 0.0	18.0 ± 0.5	1.4 ± 0.0
**K4**	99.6 ± 0.0	95.7 ± 0.3	10.6 ± 0.2	9.7 ± 0.0
**K5**	99.1 ± 0.0	91.3 ± 0.2	11.9 ± 0.1	11.8 ± 0.1
**K6**	98.9 ± 0.1	91.8 ± 0.1	10.2 ± 0.0	12.4 ± 0.1
**K7**	98.8 ± 0.2	92.8 ± 0.1	16.8 ± 0.2	9.7 ± 0.0
**K8**	98.7 ± 0.2	93.0 ± 0.0	17.0 ± 0.1	9.8 ± 0.1
**K9**	97.8 ± 0.3	93.1 ± 0.1	17.3 ± 0.1	10.6 ± 0.2

**Figure 10:**
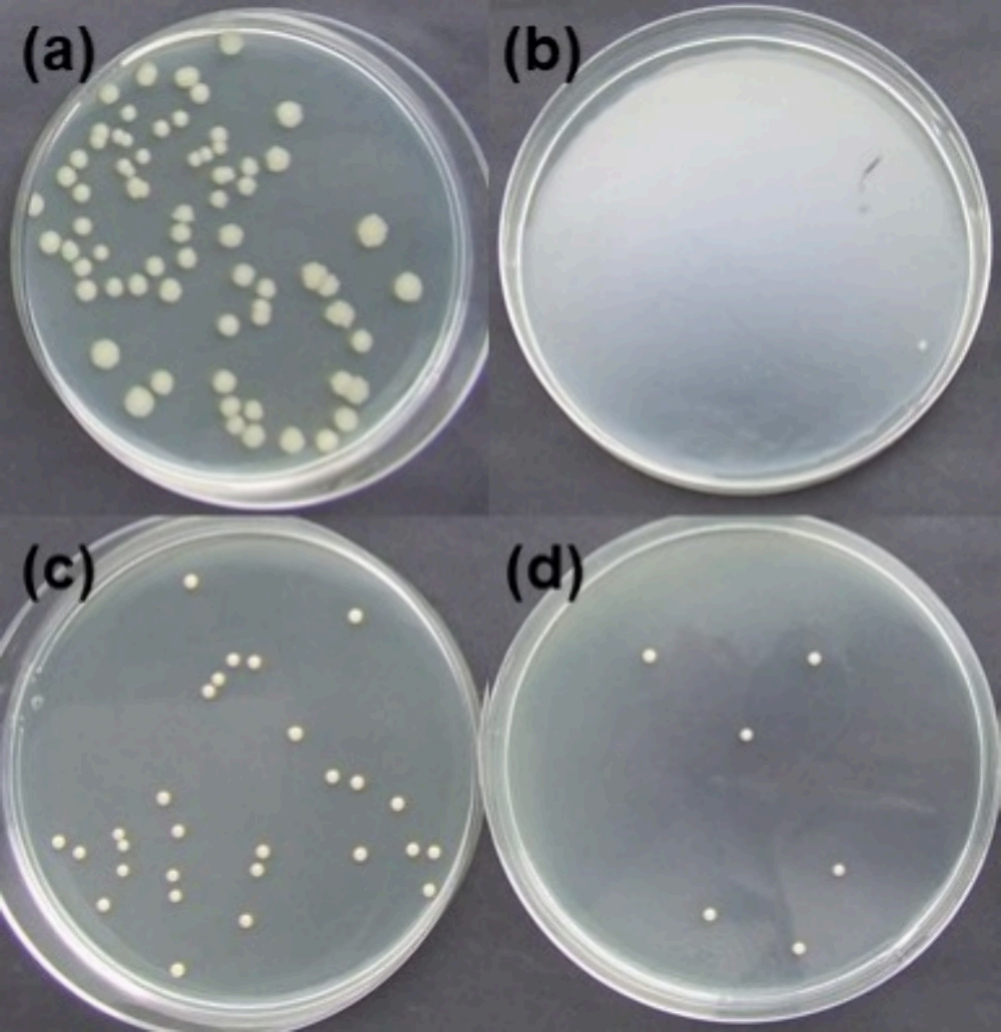
Plating of bacterial suspensions on nutrient media after 1 h incubation of inoculated polymer films: polymer **K3** with *E. coli* (a), polymer **K0** with *E. coli* (b), polymer **K3** with *S. aureus* (c), and polymer **K0** with *S. aureus* (d).

Antibacterial activity against *S. aureus* and *E. coli* was in the range of 96.4–99.6% after 1 h of contact. Polymers exhibited higher antibacterial activity against Gram-negative bacteria *E. coli* (97.8–99.6%) compared to Gram-positive bacteria *S. aureus* (96.4–95.7%). The reason for that was *S. aureus* had a higher resistance to antibiotics and antimicrobial agents compared to *E. coli* due to its capacity to adapt to different environmental conditions and remain viable under unfavorable conditions.^49^ The other reason for that might be the thinner cell wall of *E. coli*, compared to *S. aureus*, since the fatter cell wall protects *S. aureus* from antibacterial materials.^50^ In all cases, the antibacterial activity of polymers reduced with the increase in the amount of hydroxyl groups in the polymer structure. It is known that hydroxyl groups can affect the antibacterial activity of polymers^51^ and, in the case of PGTE-based polymers, reduce it. Because of that, PG-containing polymers **K1–K3** show the lowest antimicrobial activity against *E. coli* (96.2–98.1%) and *S. aureus* (90.3–90.4%). The antibacterial activity of polymers **K0–K9** after 1 h of contact is higher than that of vanillin alcohol diglycidyl ether-based polymers, which reduced the viability of *E. coli* by about 50–70% and *S. aureus* by about 30–60% after 1 h of contact. However, these polymers showed a 100% reduction of microbial spores of both bacteria.^41^ These results indicate that after 24 h, which is the standard time for antimicrobial testing, the antibacterial activity of polymers **K0–K9** could be expected to reach 100%. Antibacterial properties expand the application areas of these polymers to functional antibacterial coatings, which could prevent the spread of bacteria and extend the usage time of products. As an example, the bacterial suspension plating on nutrient media after inoculation and 1 h incubation of polymers **K0** (higher antibacterial activity) and **K3** (lower antibacterial activity) is presented in Figure 10.

**Figure 11:**
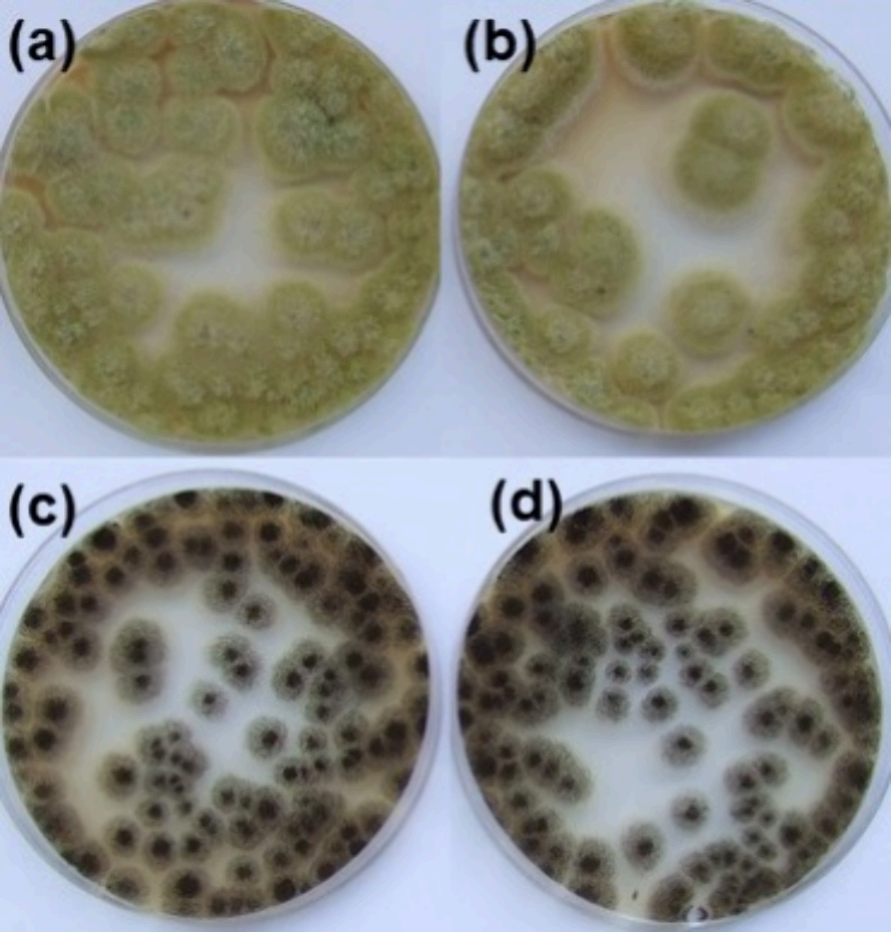
Plating of fungal suspensions on nutrient media after 1 h incubation of inoculated polymer films: polymer **K4** with *A. flavus* (a), polymer **K9** with *A. flavus* (b), polymer **K4** with *A. niger* (c), and polymer **K9** with *A. niger* (d).

The antifungal activity of PGTE-based polymers was tested against two microscopic fungi, *A. niger* and *A. flavus*. All synthesized polymers showed very similar antifungal activity against *A. niger* with less than 4% difference between polymers, and no correlation between antifungal activity and polymer structure was observed. Fungi are more resistant to antimicrobial materials than bacteria because of their complex structure. Chitin, located in the cell walls of fungi, protects them from the antimicrobial action of polymers and it takes more time to reduce their viability.^52^ Vanillin alcohol diglycidyl ether-based polymers only slightly reduced the viability of *A. flavus* (about 35%) and *A. niger* (about 40%) after 1 h. However, after 24 h, the viability of both fungi was reduced to 98.3–100.0%, indicating that for better antifungal activity a longer period of time is needed.^41^ The only exception was polymer **K3**, which reduced the viability of *A. niger* only by 1.4%. The reason for that might be spore germination and the formation of vegetative cells from spores under favorable conditions. As an example, the fungal suspension plating on nutrient media after the inoculation and 1 h incubation of polymers **K9** (higher antifungal activity) and **K4** (lower antifungal activity) is presented in Figure 11.

### 3.7 Microimprint Lithography Testing

**Figure 12:**
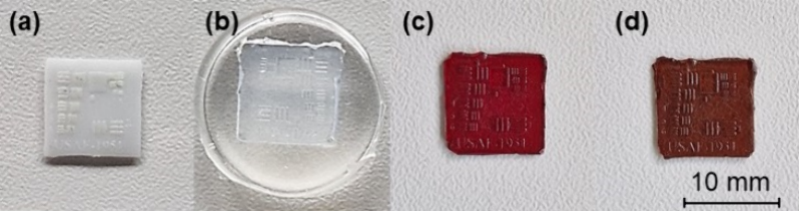
Pictures made during the microimprint lithography test: 3D printed target (a), soft PDMS mold (b), and replicas made from resins **K1** (c) and **K9** (d).

Photocurable resins were tested in microimprint lithography, which is one of the fastest and least complicated methods for the production of widely used microelectronic devices.^16^ Pictures taken during the microimprint lithography test are presented in Figure 12. Figure 12a presents the 3D printed target. The thicknesses of the lines were 67–70 μm (thin lines) and 220–230 μm (thick lines). Both replicas of **K1** and **K9** corresponded to the PDMS mold and were able to keep its shape, including all unique features (numbers, letters, lines). Resins **K1** and **K9** show great potential to be used in microimprint lithography.

## 4 Conclusions

For the first time, biobased photopolymers have been synthesized from PGTE with and without different comonomers, PG, ISO, and CHDM, which were chosen to increase the flexibility and reduce the brittleness of the resulting polymers. The neat PGTE polymer showed the highest value of the storage modulus (330.76 MPa) and the fastest photocuring time (*t*_gel_ = 325 s). The addition of comonomers reduced the storage modulus down to 15.42–314.01 MPa and increased the gel time up to 434–861 s. The tensile test showed that the addition of comonomers from 10 to 30 mol % increased the elongation at break from 0.9 to 1.89–4.51% but reduced the Young’s modulus from 20.05 GPa to 14.21–18.23 GPa and the tensile strength from 292.00 MPa to 132.62–234.54 MPa. Polymers prepared with CHDM were the most flexible with the highest elongation-at-break values (3.84–4.51%). All polymers showed thermoresponsive shape-memory properties and were able to maintain a temporary shape below their *T*_g_ and return to their permanent shape when the temperature was again increased above their *T*_g_. Also, all polymers exhibited high antimicrobial activity against *Staphylococcus aureus* (90.3–96.4%) and **Escherichia coli** (97.8–99.6%) even after 1 h of contact with bacteria, but their antifungal activity against *Aspergillus flavus* (10.6–18.0%) and *Aspergillus niger* (1.4–13.3%) was significantly lower after 1 h of contact with fungi. During microimprint lithography testing, all polymers were able to replicate the features of the 3D printed target precisely with no visible gaps or deformations. Synthesized biobased photopolymers have a wide range of properties, making them potential candidates for the production of coatings, biomedical devices, or flexible electronics that lack biobased alternatives to petroleum-based photopolymers. The composition with CHDM was the most promising due to fast photocuring and the highest flexibility. Nevertheless, other compositions are also valuable for applications where flexibility is less important, for example, as functional coatings.
